# Maternal and neonatal implementation for equitable systems. A study design paper

**DOI:** 10.1080/16549716.2017.1346925

**Published:** 2017-08-29

**Authors:** Elizabeth Ekirapa-Kiracho, Moses Tetui, John Bua, Rornald Muhumuza Kananura, Peter Waiswa, Fred Makumbi, Lynn Atuyambe, Judith Ajeani, Asha George, Aloysuis Mutebi, Ayub Kakaire, Gertrude Namazzi, Ligia Paina, Suzanne Namusoke Kiwanuka

**Affiliations:** ^a^ Makerere University School of Public Health (MakSPH), Makerere University, Kampala, Uganda; ^b^ Epidemiology and Global Health Unit, Department of Public Health and Clinical Medicine, Umeå University, Umeå, Sweden; ^c^ Global Health Division, Karolinska Institutet, Stockholm, Sweden; ^d^ Maternal and Neonatal Health Center of Excellence, Makerere University School of Public Health (MakSPH), Makerere University, Kampala, Uganda; ^e^ Department of International Health, Johns Hopkins Bloomberg School of Public Health, Baltimore, MD, USA; ^f^ School of Public Health, University of the Western Cape, Robert Sobukwe Road, Bellville 7535, Republic of South Africa

**Keywords:** MANIFEST - Maternal and Neonatal Implementation for Equitable Systems Study, Participatory action research, maternal and neonatal health, low-income countries, implementation science, community health workers, health insurance, sustainability and local capacity

## Abstract

**Background:** Evidence on effective ways of improving maternal and neonatal health outcomes is widely available. The challenge that most low-income countries grapple with is implementation at scale and sustainability.

**Objectives:** The study aimed at improving access to quality maternal and neonatal health services in a sustainable manner by using a participatory action research approach.

**Methods:** The  study consisted of a quasi-experimental design, with a participatory action research approach to implementation in three rural districts (Pallisa, Kibuku and Kamuli) in Eastern Uganda. The intervention had two main components; namely, community empowerment for comprehensive birth preparedness, and health provider and management capacity-building. We collected data using both quantitative and qualitative methods using household and facility-level structured surveys, record reviews, key informant interviews and focus group discussions. We purposively selected the participants for the qualitative data collection, while for the surveys we interviewed all eligible participants in the sampled households and health facilities. Descriptive statistics were used to describe the data, while the difference in difference analysis was used to measure the effect of the intervention. Qualitative data were analysed using thematic analysis.

**Conclusions:** This study was implemented to generate evidence on how to increase access to quality maternal and newborn health services in a sustainable manner using a multisectoral participatory  approach.

## Background

Uganda is one of the low-income countries that were unable to meet the fifth Millennium Development Goal (MDG). This was partly due to weaknesses in the health system, limited health financing and inadequacy of resources to cater for its growing population. Utilization of maternal health services is constrained by demand-side factors, such as: inadequate birth preparedness, lack of affordable transport to facilities, financial inaccessibility to cover care costs, preference for alternative traditional providers for prenatal or delivery care services, and negative attitudes towards health facilities [1–3]. Supply-side factors such as inadequate health staffing levels, hostile attitudes of health workers towards clients and suboptimal availability of medicines and equipment further compromise the quality and utilization of health services in Uganda [4,5].

In recognition of the persistently poor maternal and neonatal health outcomes in especially rural parts of Uganda, between 2008 and 2010, Makerere University School of Public Health (MakSPH) implemented two trials, the Safe Deliveries Study (SDS) and the Uganda Newborn Study (UNEST), in which local innovations to increase access to maternal and child health services were tested. Both SDS and UNEST had positive effects on increasing access to care and improvement in the care practices and survival of newborns, respectively [1,2]. The SDS used service and transport vouchers in Kamuli and Pallisa districts to increase access to quality maternal health services using a quasi-experimental study design [1]. While UNEST used community health workers (CHWs), also known as village health teams (VHTs), to encourage households to prepare for birth using birth plans and to promote newborn survival for low birthweight babies using the kangaroo mother care strategy, in addition, it strengthened the quality of services by providing refresher training, support supervision and purchasing equipment and supplies to complement what existed at the facility [2].

Although the SDS and UNEST were designed and implemented with the participation of the districts, their participation was limited and the projects were largely implemented by an external team. The use of these parallel designs denied the projects key opportunities to strengthen the existing system and therefore decreased chances of scale-up and sustainability within the system. Such opportunities include: working with local communities to identify barriers and solutions to accessing maternal and newborn health services, and working with local managers and leaders as the lead architects and implementers of the interventions. These opportunities are thought of as critical in drawing from local expertise, resources and building local management capacity, which are key ingredients for the scale-up and sustainability of initiatives [6–10]. In addition, each of the projects faced some challenges that needed to be addressed to improve their effectiveness. In the SDS, the link between the community and the health facility was weak, making it difficult to identify mothers and newborns that had complications, Quality of care at some of the health facilities was also poor. Furthermore, the project did not target challenges in newborn care practices. In the UNEST study, challenges included inability to assist mothers, who were unable to afford transport to the health facilities; and late attendance of the first postnatal care (PNC) visit by CHWs.

In an attempt to solve these challenges, MakSPH employed a Participatory Action Research (PAR) approach to engage local communities in generating feasible solutions to challenges faced by the SDS and UNEST study. The PAR approach advocates for the partnership of researchers with the research subjects or communities in order to challenge the existing undesirable conditions [11,12]. The approach promotes the empowerment of local stakeholders to identify problems and solutions to the problems, in addition to promoting learning from their experiences [13]. This has been shown in especially community-level programmes to develop local capacity and increase sustainability chances [12]. The use of this approach to address system-level challenges in the health sector is increasingly gaining popularity, although it still remains rare [14–16].

The Maternal and Neonatal Implementation for Equitable Systems (MANIFEST) study, hence, sought to combine but modify the original SDS and UNEST studies to develop more sustainable improvements in maternal and newborn care by actively engaging local stakeholders. Community mobilization strategies were aimed at supporting locally organized, financed and monitored transport systems, rather than relying on vouchers that are externally initiated and funded. The linkages between the community and the health facilities were strengthened in order to ensure follow-up and identification of community deliveries and referral. Quality of care was improved using non-financial incentives, which were thought to be more sustainable than financial incentives.

## Study aim and objectives

The study aimed at improving access to quality maternal and neonatal health services in a sustainable manner by using a participatory action research approach.

The specific objectives of the study were:

- to assess the effect of the mobilization and empowerment strategies on birth preparedness and knowledge of maternal and newborn danger signs within the communities;
- to assess the effect of the intervention on availability of community transport systems for maternal health;
- to evaluate the effect of the intervention on the quality of maternal and neonatal health services provided at the health facilities;
- to assess the effect of the intervention on the utilization of maternal health services and newborn care practices among pregnant and newly delivered women; and
- to assess the incremental cost of the intervention.

## The theory of change of the MANIFEST intervention

**Figure 1.**
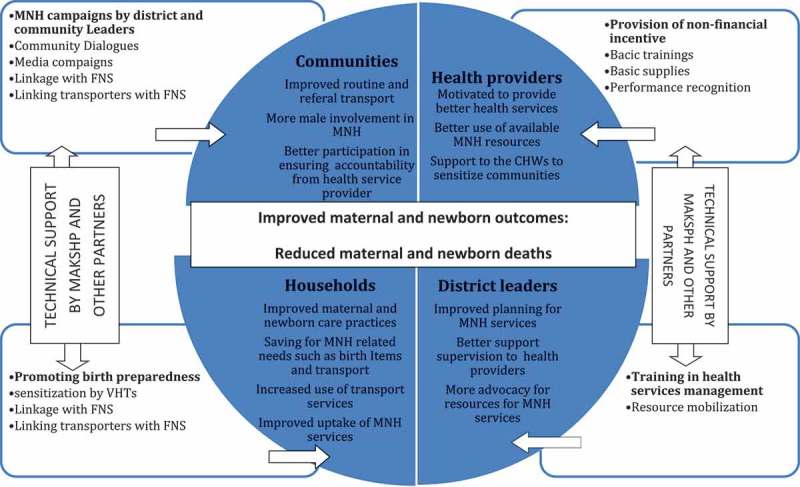
An illustration of the intervention’s casual pathways.

We hypothesized that households, communities, health workers and district-level managers could work together towards improving the lives of mothers and newborns and ultimately reducing maternal and newborn deaths. Our key assumptions were: improved birth preparedness practices would increase demand for maternal and newborn health (MNH) services; linkages between households, financial social networks and transporters would increase utilization of MNH services; motivation of health workers and improvement of their skills would improve quality of MNH services; improved management capacity of the District Health Management Team (DHMT) and health facility managers would lead to sustainable improvements in the quality of MNH services; and stakeholder engagement and sharing of lessons learned would lead to better MNH practices and policies. The theory of change is depicted in Figure 1.

At the household level, we assumed that as knowledge and awareness about MNH increases and the ability of household members to prepare for birth, by attending antenatal care (ANC), saving for birth items and for transport to facilities, is improved, then uptake of maternal and newborn care practices and facility services would increase. The community mobilization and sensitization to increase knowledge and awareness about MNH and birth preparedness was done using a combination of methods.

Lack of transport in these communities was a key barrier to maternal and newborn care services uptake. We therefore encouraged households to link up with transporters through existing financial social networks. At the onset of the study in 2013, the quality of health services, which is critical for health care utilization and uptake of maternal health services, was notably poor. Inadequate capacity of health workers and poor oversight were some of the factors that contributed to these poor-quality services [1,17]. We therefore believed that improving health worker motivation and improving skills of health workers through the provision of non-monetary incentives, such as skills-based training, recognition, mentorship and supportive supervision, would help to improve the quality of services. In addition, as noted by the World Health Organization [18], managerial skills are important for improving the quality of services at the facility level and their subsequent uptake. We therefore planned to support district and facility-level managers to attend a distance health management course. We believed that through the knowledge gleaned from the management course, as well as overseeing the implementation of MANIFEST, the district managers and those in charge of facilities would accumulate the expertise needed to plan for services, manage district resources for health and improve health services quality.

At the institutional level, we planned to build partnerships with the communities, districts and national stakeholders. We believed that these partnerships would enable learning and facilitate the uptake of best practices for MNH services emerging from MANIFEST at both national and community levels, all of which would ultimately contribute towards better services and improved health outcomes for more mothers and newborns.

## Methods

### Study site

The study was implemented in Pallisa, Kibuku and Kamuli Districts, which are mainly rural, where the SDS study that used vouchers for transport, ANC, delivery and PNC services was implemented previously [1]. Pallisa has two health subdistricts (HSDs), Kibuku has one HSD and Kamuli three. A health subdistrict is an administrative zone within the district. The three districts had a total population of 1,075,242. Kibuku has a population of 202,033, Pallisa 386,890, while Kamuli has 486,319 [19].

A DHMT heads the health system at the district level. The DHMT is located at the district headquarters within a department of health headed by a district health officer, with a team of about 15 administrative and management staff. At district level, the higher-level health facilities comprise a hospital and a health centre (IV), which provide inpatient and outpatient care as well as surgical services. The hospital serves the whole district population, which is about 335,536 on average while the health centre (IV) serves approximately 126,586 people [20]. The lower-level facilities consist of a health centre (III) and health centre (II). The health centre III has a general outpatient clinic, admits patients and has a functional maternity and laboratory service, while the health centre (II) provides mainly outpatient services. The health centre III serves an average population of 26,785, while the health centre II serves an average population of 5,057 (20). The lowest level of care in the district health system is the health centre I (HCI). The HCIs are voluntary service structures, which provide mainly mobilization, sensitization and outreach activities through VHTs. The VHT is ideally comprised of five volunteers per village who are responsible for providing these services at their respective villages. At village level, the average population size is estimated at 650 persons [20]. The health service infrastructure in the study area comprised a total of 104 health facilities; 33 in Pallisa, 17 in Kibuku and 54 in Kamuli.

### Study design and population

The intervention was implemented using a quasi-experimental design. We had three HDSs in which the intervention was implemented and two comparison HSDs. Pallisa, Kibuku and Buzaya (Kamuli) HSDs were taken as intervention areas, whereas the HSDs of Bugabula (Kamuli District) and Butebo (Pallisa district) were the comparison areas. The intervention package was provided only to the intervention arm. However, some aspects of the intervention such as home visits by the VHTs were at times provided by other non-government organizations (NGOs) in the comparison arm as well. The package offered by these VHTs was, however, often different from that provided by the MANIFEST study. They may also have received the radio messages that were aired over the radio, as well as some aspects of the support supervision that was done by the district team in both intervention and comparison areas as part of routine supervision of health facilities in the district.

The study population were women of reproductive age (15–49 years) and men of reproductive age, health workers and district health officials, transporters and members of community savings groups, subcounty chiefs and community opinion leaders.

### Description of the MANIFEST intervention

The intervention had two phases: the preparatory phase and the implementation phase.

### Preparatory phase

The preparatory phase comprised of the development and adaptation of materials and tools, setting up of implementation structures, selection and training of CHWs.

#### Development and adaptation of materials and tools

The materials for training CHWs and their supervisors were adapted from those used by the Ministry of Health and the UNEST study. The kits that were used by CHWs during home visits were also adapted from the UNEST study. These kits included registers, referral forms, birth plan forms, family cards and report forms. The VHTs were given a bag for carrying these materials and a T-shirt for identification purposes.

Training materials about safe delivery, newborn care and resuscitation were adapted from the existing Ministry of Health (MoH) training and demonstration materials. The performance assessment tools for health facilities and health workers were also adapted from the existing standard assessment tools developed by MoH. The DHMTs in collaboration with the MakSPH research team were responsible for preparing radio spots messages and talk shows in respective local languages.

#### Setting up implementation structures at district level and community level

The district level stakeholders played a lead role in the implementation of the project through the district-, subcounty- and parish-level committees. The committees at district and subcounty level were composed of existing management committees, to which additional members such as political and religious leaders were added. The parish-level committee, on the other hand, was composed of CHW representatives from each village. After setting up the community-level implementation committees, they were oriented about their roles and expected outputs by the DHMT members.

#### Selection of CHWs

The DHMTs across the three districts selected two VHTs out of the existing community volunteers working with the health department. In the villages that did not have volunteers, the local village leadership organized meetings, in which two volunteers were selected. The selection of VHTs was guided by the MoH guidelines for VHT selection. There were 27 subcounties with 847 villages in the three districts (514: 346 in Pallisa and 244 in Kibuku) from which 1,694 CHWs were selected.

#### Training of CHWs

The CHWs received a four-day training by the district trainers. The district trainers were trained and supported by national VHT trainers. The training was conducted in local languages and focused on birth preparedness, maternal and newborn danger signs, referral of pregnant women and newborns, how to conduct home visits, and how to give health education talks. The CHWs also received training about family planning.

### The implementation phase

The implementation phase was designed to have two main components: community empowerment for comprehensive birth preparedness and health provider and management capacity-building.

#### Community empowerment for comprehensive birth preparedness

This component had the following activities; home visits by community health workers, community mobilization and sensitization using community dialogue meetings and radios and linking communities with financial social networks and local transport providers.

#### Home visits by the community health workers

The trained CHWs conducted two home visits during pregnancy and two after delivery. They also counselled mothers on essential maternal and newborn care practices, safe delivery and birth preparedness. The CHWs identified women and children with danger signs and those identified were refered to the health facility for futher screening and care.

#### Community mobilization and sensitization

The CHWs, with the assistance of local council leaders, mobilized community members for sensitization and dialogue meetings, while the district health team used radio spot messages and talk shows to sensitize the community. The sensitizations and dialogue meetings addressed issues concerning birth preparedness, men’s role in maternal and child health care, joining saving groups, organizing transport to the health units, PNC and family planning.

The community dialogues were conducted on a quarterly basis and were facilitated by CHWs and resource persons within the community. The radio talk shows and spots, on the other hand, were conducted on a monthly basis and daily for specific time periods during the project implementation, respectively. The role of the radio sensitizations was majorly to reinforce the messages discussed in the community dialogues and VHT home visits.

#### Supervision of CHWs

The CHW supervisors (health assistants and health workers) conducted directly observed supervision of CHWs in the villages once a month to reinforce their skills. In addition, the CHWs had monthly meetings with their supervisors to support them with report writing, problem-solving and reinforcing skills. The CHW leaders at parish level (one per parish) also mobilized and oversaw the work of fellow CHWs in each parish. On a quarterly basis, a combined meeting of CHWs, supervisors, and members of the DHMT to share work experiences and reinforce skills was undertaken. The CHWs submitted their reports to their parish-level representative, who in turn submitted them to the health assistant for record keeping on a monthly basis.

#### Linking communities with financial social networks and transporters

Community development officers, VHTs and local council leaders mobilized community members at village level and sensitized them to form or join existing saving groups. These saving groups provided social protection in terms of financial benefits that cater for both domestic and health care needs of the members. Communities were also encouraged to form agreements through their savings groups with local transporters to ease transportation to health facilities. The agreement would garantee the transporter payment through the savings groups.

### Health provider and management capacity-building

This component had three main activities: (1) training and skills building for health workers and managers, (2) provision of basic support, and (3) recognition of good performance.

#### Training and skills building

The DHMT received training about planning and management of health services, including management of medical logistics, support supervision and mentoring of staff. This was done through two kinds of courses: (1) a short course in health services management that targeted mid- and lower-level health managers, and (2) a postgraduate diploma in monitoring and evaluation for the district health officers. In addition, these managers were actively involved in the implementation of the MANIFEST project as a means of reinforcing their skills, as well as building any specific management skills in order to foster quality and continuity as noted earlier. The general frontline health workers, on the other hand, received a work-based training in the management of obstetric emergencies and newborn care given by external experts. In addition, mentorship and support supervision were provided to reinforce these skills.

#### Provision of basic support

The participating health facilities received equipment for conducting safe deliveries and neonatal resuscitation to improve the quality of existing services. The district health office selected the items and equipment that was to be purchased by the health facilities. The equipment provided to each of the districts was valued at approximately $3,000 and included delivery beds, newborn resuscitation kits, delivery kits, oxygen cylinders and vacuum extractors to aid difficult vaginal childbirth.

#### Recognition of good performance

The performance of the participating health facilities and health workers was assessed on a quarterly basis using the standard MoH assessment tools. The best two performing facilities and health workers, and the most improved facility per district in a period of six months (two quarters), were given recognition awards by the DHMT and the project. This was in order to motivate them, maintain their performance, and also inspire others.

### Sample size estimation

#### Qualitative data

The participants for qualitative data were selected purposively depending on the research question being investigated and the saturation and maximum variation principles were employed in deciding the sample size. Focus group discussions (FGDs) were done with women and men of reproductive age. The FGDs were stratified according to the community sociodemographic characteristics (hard to reach, accessible, rich and poor communities). In each of the strata, three FGDs comprising 8–12 people were conducted at baseline and endline. Twenty-eight key informant (KI) interviews were done with health workers, health managers, members of the district health team and politicians. Table 1 provides detailed information about the number of FGDs and KIs that were conducted in the intervention and comparison arms.

**Table 1.** Sources of focus group discussions and key informant interviews conducted at each point of evaluation data collection (baseline and endline).

Data collection method	Number in the intervention area	Number in the comparison area	Respondent category
Key informant interviews (Community leaders)	12	6	Local council III Chairpersons, Super Village health team members, Community development officers, Health assistants, Savings group leaders, Secretary for health and Subcounty chiefs
Key informant interviews (Health facility managers and staff)	6	4	Health facility staff and managers from both private and public health facilities that benefited from the mentorship programme
Focus group discussions	9	6	Women who delivered in the last 12 months and their partners
Total	27	16	

#### Quantitative data

The sample size for the household survey with women of reproductive age was based on the intervention’s assumption that, after three years (2013–2015) of implementation, skilled deliveries in the intervention area of Kibuku, Pallisa and Kamuli districts would increase from 38% to 58%, 62% to 72% and 68% to 78%, respectively [21]. The sample size was determined using a two-sided *Z*-test of the difference between proportions (Equation 1) with 80% statistical power, a 5% significance level, and 1.5 design effect, which resulted in a sample size of 2,293 women.

Sample size determination formulae

(1) $$n=\frac{{\left(Z_{\alpha /2}+Z_{\beta }\;\right)}^{2}\left((\pi_{1}\left(1/\pi_{1}\right)+\pi_{2}\left(1/\pi_{2}\right)\right)}{{\left(\pi_{1}-\pi_{2}\;\right)}^{2}}$$

The sample size for the household survey with men of reproductive age was dependent on the assumption that their knowledge on maternal danger signs was 90%, in line with a study done in Kenya [22]. Using the Kish Leslie sampling formula at 5% level of significance, precision and non-response rate, a sample of 218 men per study arm in each of the districts was calculated.

The sample size for the exit interviews was based on the assumption that the level of satisfaction and the quality of MNH services in the three districts would increase by 15% (from 50% to 65%) in the intervention area over the three-year period (2013–2015). The sample size was determined by a two-sided *Z*-test of the difference between proportions (Equation 1) with 80% power and a 5% significance level. From the calculation, a sample of 780 mothers was calculated for the baseline and endline survey.

### Data collection methods and tools

The evaluation data were collected using both qualitative and quantitative methods. Data were collected before project implementation (baseline), during project implementation (midterm), and at the end of the project implementation (endline). Table 2 details the different methods of data collection and sources used for each of the study objectives.

**Table 2.** Details of data collection methods and sources.

Research objectives	Indicators	Data collection methods	Respondents
To assess the effect of the mobilization and empowerment strategies on birth preparedness and knowledge of maternal and newborn danger signs within the communities	Percentage change in saving for maternal and newborn health Percentage change in knowledge of maternal and newborn danger signs	Baseline and endline surveys	Women of reproductive age
		Key informant interviews	Men
		Focus group discussions	VHT reports
		Routine	Representatives
To assess the effect of the intervention on availability of community transport systems for maternal health	Percentage change in the use of organized community transport Availability of community transport in each subcounty	data collection	from savings groups
			Community development officers
			Local transporters
To evaluate the effect of the intervention on the quality of maternal and neonatal health services provided at the health facilities	Changes in the use of standards and protocols Proportion of health facilities with skilled health providers for maternal and newborn health Perception of the community on the qulity of care given at the health facilities Percentange of health workers with skill in management of maternal and newborn complications	Baseline and endline surveys	Women of reproductive age
		Key informant interviews	Health workers
		Focus group discussions	Facility records
		Review of facility records	Supervision and mentorship reports
		Review of supervision and mentorship reports	Community leaders
		Health facility assessments	District health team members
		Health worker survey	Political leaders of the district
		Exit interviews	Health managers
		Document reviews	Stakeholder
To assess the effect of the intervention on the utilization of maternal health services and newborn care practices among pregnant and newly delivered women	Percentage changes in health facility delivery, ANC attendance, and PNC attendance Percentage change in delayed bathing, immediate breastfeeding, and putting nothing on the newborn cord	Meeting minutes	meetings
To obtain the incremental cost of the intervention	Cost of intervention dissagregated by different interventions	Review of financial records	Financial records

### Qualitative data collection

#### Selection of study subjects

The FGDs and KI interviews were done at community level. The participants were identified purposively with the help of local council officials who also set-up the venue ideal for the discussion in their community.

#### Data collection methods and procedures

Key informant interviews and focus group discussions were used to collect data about the perceived quality of maternal and newborn services, factors that affect delivery of MNH services and saving practices. They were also used to obtain the stakeholders’ perception about different components of the intervention. Stakeholder meetings were also used to collect information about the intervention. KI guides, FGD guides, personal memos, observation checklists, meeting reports were used to collect the qualitative data.

#### Data collection procedures

Trained research assistants moderated the FGDs and KI interviews and documented the process. All the interviews were audio-recorded and transcribed later with the help of research assistants. On the other hand, the district health team members, assisted by the project team, facilitated the stakeholders’ meetings at district and subcounty level. The participants’ observations and key issues noted were documented in memos and meeting minutes. All the study documents were stored on a web-based database with access restricted to only project team members.

#### Quality assurance

To ensure the quality of data collected the following measures were considered. A public health qualitative expert trained and supervised the research assistants who conducted the qualitative interviews. All the data collection instruments including FGDs and interviews were pretested in one of the districts that was considered to have similar community characteristics as the study implementation districts. KI interviews and FGDs were audio-recorded in order to minimize loss of information. Regular review meetings were conducted to facilitate reflection on personal level observations of the study team and other stakeholders involved.

#### Data analysis and management

The qualitative data collected before and after the implementation of the intervention were transcribed and reconciled with notes recorded during the interviews and then analyzed using thematic and content analysis [23]. Reading and rereading of the data to facilitate familiarization with the data preceded the coding of the data. The coding process was both inductive and deductive guided by the objectives of the study. Different levels of abstraction were then applied to the codes to develop further the analysis process depending on the method used for each specific objective. Where appropriate, theoretical frameworks were used to facilitate the analysis and interpretation of the findings. An active process of writing memos and reflection also facilitated the analysis processes.

Reviewing of the processes by the different researchers was conducted to ensure study worthiness. For example, in the thematic analysis, the themes were reviewed to ensure that there was internal homogeneity and external heterogeneity between the themes.

### Quantitative data collection

#### Selection of study subjects

A two-stage sampling was applied per district for each of the study areas during the household survey. First, the villages were selected using probability proportionate to size sampling techniques, and thereafter all households were listed in order to identify eligible study participants. Women whose pregnancies were terminated before 20 weeks and women who were not residents and had not stayed in the community for at least one year were excluded from the study.

Regarding the health facility exit interviews, all women who delivered from the health facilities were interviewed on discharge until the predetermined sample size was realized. Women aged less than 18 years but who were married and could provide individual consent were also included. On the other hand, those with severe illnesses at the time of the survey and those that refused to consent were excluded.

#### Data collection methods and tools

##### Household surveys

Household surveys were conducted among women of reproductive age and used to collect information about knowledge of maternal and newborn danger signs, utilization of maternal health services and birth preparedness. A household survey was done among men of reproductive age to obtain information about knowledge of maternal and newborn danger signs.

##### Exit interviews

Client exit interviews were done to assess client satisfaction wth maternal and newborn health services.

##### Health facility assessment and records review

We assessed the quality and availability of services in all 43 health facilities that were providing maternal and newborn services from both intervention and comparison areas. This assessment was used to collect information on the availability of staff, essential equipment and drugs for maternal and newborn health services. In addition, we undertook monthly review of records on maternal death, newborn death, stock out of drugs, still births, health facility delivery, management of obstetric and neonatal complications, ANC and PNC attendance from all the 43 health facilities.

The above quantitative data were collected using electronic health management information forms, VHT community records, structured questionnaires and observation checklists.

##### Data collection procedures

Trained research assistants collected data during the household and health facility exit interview surveys. They were supervised by senior researchers. During data collection, data editors checked all the completed questionnaires for errors and missing information. Any error identified was verified and corrected immediately by the research assistants while in the field. The research assistants also collected the health facility assessment information. This was collected by observing the availability of equipment/drugs under the guidance of the health facility in-charge. The health facility records on health facility delivery, ANC attendance, PNC attendance, management of complications, maternal death, newborn death and stillbirths were collected from the health facility maternity register.

##### Quality assurance

To ensure the quality of data collected the following measures were considered: a data collection manual which stipulated what was to be done during sampling, data collection, entry, storage and management was developed and utilized. A team of medical doctors, a statistician, and public health specialists trained the research assistants. The field supervisor sampled and re-interviewed some respondents each day, in order to check for consistency and accuracy of the information being collected. All the data collection instruments were pretested in one of the districts that was considered to have similar community characteristics as the study implementation districts. A study advisory group, as well as the research partners for the Future Health System (FHS) consortium, provided regular oversight of the implementation of the study and provided advice.

##### Data analysis and management

The entered data were cleaned and then analysis was undertaken. A number of analysis techniques were used to anwser the different objectives. These included descriptive statistics, regression and multivariate analysis techniques such as the difference in difference analysis and logistic regression. The results of this intervention are provided in the different papers that are part of this supplement.

### Study strengths and limitations

The MANIFEST study had several strengths. It comprised a multisectoral intervention package, which allowed it to address different demand- and supply-side constraints that hinder acess to MNH services. This intervention was implemented using a participatory action approach that is lauded for its potential to promote local ownership and participation of local stakeholders. The study also used both quantitative and qualitative data collection methods and this enabled us to have a quantitative assessment of the effect of the intervention with indepth explanations of how different factors influenced the intervention.

Although the experimental design (quasi-experiemental) used allowed us to have a comparison area and to assess the contribution of the intervention, it did not allow us to assess the separate effects of each component of the intervention package. The second limitation is that the participatory approach used to implement the research is dependent on local stakeholders who may not have all the required skills, and this may have affected the intervention implementation efficiency. However the study team provided technical support through out the study. Another limitation was that there was a risk of contamination, because the comparison area is part of the district where the intervention is and staff sometimes get transferred from the intervention area to the comparison area. Another source of contamination is the radio which was used as a channel for communicating some messages. The listenership for the radio includes community members in the comparison area.

## Conclusions

This study was implemented to generate evidence on how to increase access to quality maternal and neonatal health services in a sustainable manner [14]. The use of the PAR strategy has been documented to have the potential of strengthening the health system which is essential for sustainability of interventions [24]. In this supplement, we share a special issue of 10 different papers of which this paper is part. The rest of the nine papers share specific results [25–32]. In addition to this design paper, there is one more methodological paper that describes the PAR approach in more detail [33]. Two of the papers [25,26] summarize the results of the intervention on some of the key outcomes of the study (knowledge about maternal and newborn danger signs, birth preparedness and utilization of maternal and newborn health services and newborn care practices). Two other papers describe the characteristics of saving groups and the saving practices of the community for maternal and newborn health, highlighting opportunities for promoting saving for maternal and newborn health [27,28]. Lastly, three of the papers focus on the health system interventions that were implemented [29–31]. One of them describes the use of community health workers [29], another presents the achievements made through the support supervision component of the intervention [30] and the third shows the achievements and challenges of implementing the mentorship component of the program [31]. The final paper explores factors that contribute to the retention of health workers [32]. Although interventions such as this one may build the capacity of health workers, these health workers are often not retained for long periods.

## Paper context

The use of participatory action research is still in its infancy. This design paper contributes to the methodological development on its use in implementation science health projects. It also contributes to the literature on how to spread local innovations in health care delivery, highlighting the strategies and factors that promote learning at organizational and community levels.
